# Diabetes Status After Lateral Pancreaticojejunostomy and Frey’s Procedure in Chronic Calcific Pancreatitis: An Observational Study

**DOI:** 10.7759/cureus.21855

**Published:** 2022-02-03

**Authors:** Ashok Kumar Sahoo, Narendranath Swain, Arun Kumar Mohanty, Sibabrata Kar, Nagendra Kumar Rajsamant, Santosh Kumar Behera

**Affiliations:** 1 Surgery, S.C.B. (Srirama Chandra Bhanja) Medical College and Hospital, Cuttack, IND; 2 Surgery, Jawaharlal Institute of Postgraduate Medical Education & Research, Puducherry, IND; 3 General Surgery, S.C.B. (Srirama Chandra Bhanja) Medical College and Hospital, Cuttack, IND

**Keywords:** alcohol, follow-up, insulin, clinicopathological diagnosis, drainage procedure

## Abstract

Introduction

Diabetes secondary to pancreatic diseases is commonly referred to as pancreatogenic diabetes or type 3c diabetes mellitus. This study was conducted to determine the status of diabetes mellitus after Frey’s procedure and lateral pancreaticojejunostomy (LPJ) in diabetic and nondiabetic patients with chronic calcific pancreatitis (CCP) and to discuss the clinicopathological course as well as diabetes in CCP.

Materials and methods

This study was designed as a retrospective observational study consisting of 27 patients with CCP who were surgically treated either with the pancreatic head coring Frey’s procedure or with LPJ. Surgeries were performed in a tertiary care hospital of Eastern India by a team of surgeons following the same surgical principle. The diagnosis of CCP was made by clinical and radiological evaluations. Visual Analog Scale (VAS) scoring was used perioperatively to assess pain. Postoperatively, all the patients were monitored clinically; pain scoring and relevant investigations were done depending upon subjective and objective indications. Special attention was paid to diabetic patients through frequent follow-ups and tight glycemic control. All 27 patients were followed up with at least two outpatient follow-ups.

Results

The trends in fasting blood sugar values in the LPJ group showed a small spike in the early postoperative period (two weeks) with a p-value of >0.05, and later on, it improved over 18 months of follow-up, reaching below the preoperative values (mean 109.38). On the contrary, the fasting blood glucose levels in Frey’s procedure revealed a significant spike in the early postoperative period (two weeks) with a mean sugar value of 148 mg/dl and a p-value of 0.01. The levels stayed well above the preoperative values over 18 months of follow-up. The trends in HbA1c showed marginal improvement in the LPJ group in a six-month follow-up period (p-value 0.008) from the preoperative levels. In Frey’s procedure group, postoperative HbA1c levels at three months revealed an increase, which can be attributed to the minor but significant loss of pancreatic tissue from the head, which continued to be on the higher side at the six-month follow-up. Trends in mean insulin dosage showed a significant spike in the early postoperative period (two weeks) both in the LPJ (p-value 0.01) and Frey’s procedure group (0.01); however, in the LPJ group, the insulin dose showed a reduction over the 18-month follow-up, reaching below the mean preoperative insulin dose. While in the Frey’s procedure group, the postoperative insulin dose remained higher throughout the 18-month follow-up period (p-value <0.05).

Conclusions

LPJ has got a little effect on the diabetic status of nondiabetic patients. Frey’s procedure leads to marginal deterioration of the diabetic status and increases in insulin dosage in both diabetic and nondiabetic patients.

## Introduction

Chronic calcific pancreatitis (CCP) is a common condition encountered in the eastern parts of India. It is a disease characterized by pancreatic inflammation and fibrotic injury, resulting in irreversible parenchymal damage. Diabetes secondary to pancreatic diseases is commonly referred to as pancreatogenic diabetes or type 3c diabetes mellitus [1]. It is a clinically relevant condition with a prevalence of 5-10% among all diabetic subjects in the Western population. In nearly 80% of all type 3c diabetes mellitus cases, CCP seems to be the underlying disease [2].

The causes of diabetes in CCP are multifactorial and include atrophy of the pancreas. beta-cell destruction, exocrine insufficiency, and role of incretin [3]. Surgical management for pancreatitis is reserved for patients with complications of CCP or suspected underlying carcinoma. The choice of surgery for CCP is decided based on anatomical variants of the disease, which are distinguished by the size of the main pancreatic duct [4]. 

In CCP, the most common surgical procedure done is drainage. Recently it has been found that the incidence of carcinoma in CCP is about 15-40% [5]. This suspicion of malignancy may require resection procedures and most of the patients with symptoms are diabetic at the time of surgery and are on insulin for the control of diabetes mellitus.

During Whipple’s or pylorus-preserving pancreaticoduodenectomy, the head of the pancreas is removed. Pancreatic tissue provides insulin, which is required for blood sugar control. When pancreatic tissue is removed, the body releases less insulin, and the risk of developing diabetes mellitus is high. Patients who are diabetic at the time of surgery or who have had an abnormal blood sugar level that was controlled on diet or insulin prior to surgery have a higher chance of diabetes getting worse after surgery [6].

Head resection removes about 40% of the pancreas. Whether this resection leads to diabetes or worsening of diabetes is controversial. There are reports from various parts of the world that resection worsens diabetic status. However, these studies were based on CCP patients [7].

Many patients with CCP manifest some degree of fat malabsorption, regardless of the presence of symptoms [8]. In patients with type 3c diabetes mellitus, exocrine pancreatic insufficiency is ubiquitously present. The incretin system may play a crucial role in the metabolic control of type 3c diabetes mellitus. The regulation of the beta-cell mass and the physiological incretin secretion are directly dependent on normal exocrine pancreatic function and fat hydrolysis. Drainage procedures improve the exocrine function and may favor the betterment of diabetic status and insulin use [9].

However, so far, there have been no reported studies in CCP in the tropics to prove whether there is a deterioration of diabetes following resection/drainage procedure. Hence, this study was conducted to determine the status of diabetes mellitus after Frey’s procedure and lateral pancreaticojejunostomy (LPJ) in diabetic and nondiabetic patients with CCP and to discuss the clinicopathological course of CCP and management of diabetes in CCP.

## Materials and methods

This study was designed as a retrospective observational study consisting of 27 patients of CCP who were surgically treated either with the pancreatic head coring Frey’s procedure or with LPJ from July 2014 to December 2016. The surgeries were performed in a tertiary care hospital of Eastern India by a team of surgeons following the same surgical principle. The diagnosis of CCP was made by clinical and radiological evaluations, which include a plain x-ray of the abdomen, ultrasound scan (USG), computed tomography scan (CT scan), and magnetic resonance cholangiopancreatogram (MRCP). Various radiological investigations were used to determine the pancreatolithiasis, main pancreatic duct (MPD) contour, any alteration in the parenchymal architecture like fibrosis, atrophy, and mass lesions. Visual Analog Scale (VAS) scoring was used perioperatively to assess pain [10]. The score was reported on the scale of ‘no pain’ to ‘mild to moderate pain’ to ‘intolerable or worst pain’. The patients who were excluded from the study were those who had undergone endoscopic retrograde cholangiopancreatography (ERCP) or those requiring Whipple’s partial pancreaticoduodenectomy or Beger’s procedure.

Surgical techniques

The indications of surgery were unbearable pain due to pancreatolithiasis, dilated MPD, suspicion of malignancy, failure of medical treatment, or any associated complications. The surgery aimed to remove all ductal stones, strictures, and the diseased segment of the pancreas, followed by a wide pancreaticojejunal anastomosis. The patients undergoing Frey’s procedure had to undergo opening of the MPD, removal of all calculi, and enucleation of the diseased pancreatic head in contiguity with a strictured segment of the duct of Wirsung. A rim of the pancreatic head closed to the duodenum was spared along with posterior parenchyma, with or without excising both ducts of Wirsung and Santorini. This technical modification spares the pancreatic neck and preserves the posterior capsule of the pancreatic head along with the body and tail. In both original and modified Frey’s procedures, the depths of pancreatic tissue that was cored were different. Second-generation ducts stones were also removed. Roux-en-Y longitudinal/lateral pancreaticojejunostomy ensured wide pancreatic drainage. The tissues were histopathologically studied to exclude malignancy. 

Postoperative care

All the patients were monitored clinically and through pain scoring and relevant investigations depending upon subjective and objective indications. All the patients were started on oral feeding between the fifth and seventh postoperative days and were discharged between the 10th and 23rd days. Special attention was paid to diabetic patients through frequent follow-ups and tight glycemic control.

Follow-up

All 27 patients were followed up till July 2017 with at least two outpatient follow-ups. All patients gave written informed consent for surgical treatment according to the institutional guidelines. Long-term follow-up was obtained by: a telephonic interview with the patient, a written questionnaire that was sent to the patient. In cases of rehospitalization, medical records were obtained and reviewed. Most patients needed a proton pump inhibitor or H2 receptor blocker, a pancreatic enzyme supplement. Variables examined were fasting blood sugar (FBS), postprandial blood sugar (PPBS), HbA1c and dose of insulin, the occurrence of diabetes, and the date of initiation of insulin treatment.

Statistical analysis

Data analysis was done with IBM SPSS Statistics for Windows, Version 20.0 (Released 2011. IBM Corp., Armonk, New York). Chi-square test and paired two-tailed t-test were used for data analysis. A p-value of <0.05 was considered statistically significant.

## Results

Out of a total of 27 patients, 13 patients had undergone Frey’s procedure, and the remaining 14 patients went through LPJ. The demographic characteristics of the patients are given in Table 1.

**Table 1:** Demographic Characteristics LPJ: lateral pancreatojejunostomy

Qualitative Variables		LPJ Group (14)(%)	Frey’s Group (13)(%)	P-value
Sex	Male	9(64.3)	8(61.5)	0.883
	Female	5(35.7)	5(38.5)	
Socioeconomic status	Low	11(78.6)	8(61.5)	0.333
	Medium	3(21.4)	5(38.5)	
	High	0	0	
Alcoholism	Yes	7(50)	4(30.8)	0.310
	No	7(50)	9(69.2)	
Smoking	Yes	4(28.6)	6(46.2)	0.345
	No	10(71.4)	7(53.8)	
Hyperlipidemia	Yes	4(28.6)	8(61.5)	0.085
	No	10(71.4)	5(38.5)	
Recurrent acute pancreatitis	Yes	10(71.4)	10(76.9)	0.745
	No	4(28.6)	3(23.1)	
Cambridge score	Mild	0	0	0.580
	Moderate	11(78.6)	9(69.2)	
	Severe	3(21.4)	4(30.8)	
Preoperative Oral Hypoglycemic Agents use	Yes	1(7.1)	1(7.7)	0.957
	No	13(92.9)	12(92.3)	
Postoperative Oral Hypoglycemic Agents dosage change	Yes	1(7.1)	2(15.4)	0.496
	No	13(92.9)	11(84.6)	

Among the 14 LPJ patients, nine were males and five were females. The Frey’s group comprised eight males and five females. Both groups were comparable regarding sex, with a p-value of 0.883. In total, 11 out of 14 patients belonged to a low socioeconomic background in the LPJ group, while eight out of 13 in the Frey’s procedure group were in the low-income group. Also, three out of 14 in LPJ and five out of 13 in the Frey’s procedure group belonged to the middle-income group. Both groups were comparable as there was no statistical difference in socioeconomic status (p-value 0.333). Alcohol addiction was among 50% and 30% of patients in the LPJ group and the Frey’s procedure group, respectively. Among the LPJ group and the Frey’s procedure group, 72% and 77% had a history of recurrent acute pancreatitis, respectively. Severity was assessed using the Cambridge severity score; 79% of patients in the LPJ group were categorized as moderate and 21% as severe. In the Frey’s procedure group, 69% were classified as moderate and 30.8% as severe. The baseline characteristics of both groups are depicted in Table 2.

**Table 2:** Baseline Characteristics FBS: fasting blood sugar; LPJ: lateral pancreatojejunostomy

Quantitative Variables		LPJ Group	Frey’s Group	T-Value	P-value
Weight	Mean	58.5	60.77	0.577	0.569
	SD	9.558	10.872		
BMI	Mean	22.60	23.2	0.794	0.434
	SD	3.79	3.25		
Serum amylase	Mean	40.43	61.23	1.728	0.096
	SD	27.046	35.235		
Serum lipase	Mean	40.14	59.46	1.322	0.198
	SD	41.757	33.293		
Preop FBS	Mean	125.93	122.46	0.188	0.853
	SD	51.53	43.714		
Preop HbA1c	Mean	6.829	6.623	0.445	0.660
	SD	1.1384	1.2597		
Preop insulin	Mean	15.79	10	1.084	0.288
	SD	15.423	11.916		
FBS 2 weeks	Mean	131.14	148	1.166	0.255
	SD	37.554	11.916		
FBS 3 months	Mean	123.14	135.62	1.248	0.224
	SD	24.507	27.421		
FBS 6 months	Mean	114.23	134.77	1.892	0.071
	SD	22.5	32.037		
FBS 12 months	Mean	110.77	132.38	2.318	0.029
	SD	25.2	22.262		
FBS 18 months	Mean	109.38	126.31	1.726	0.97
	SD	25.695	24.274		
HbA1c 3 months	Mean	6.871	6.908	0.100	0.921
	SD	0.8905	0.9987		
HbA1c 6 months	Mean	6.631	6.946	0.978	0.338
	SD	0.7941	0.8491		
Postop insulin 3 months	Mean	20.371	16	0.799	0.432
	SD	15.6520	12.4633		
Postop insulin 6 months	Mean	16	16.77	0.123	0.903
	SD	17.504	13.154		
Postop insulin 1 year	Mean	17.23	15.69	0.266	0.792
	SD	16.942	12.106		
Postop insulin 18 months	Mean	15.68	15.08	0.004	1.00
	SD	16.424	10.943		

The mean weight in the LPJ group was 58.5 kg with a standard deviation (SD) of 9.558, while in the Frey’s procedure group, it was 60.77 kg, with an SD of 10.87. The mean BMI was 22.6 and 23.2 in the LPJ and Frey’s procedure groups, respectively. The mean preoperative fasting blood sugar (FBS) in the LPJ group was 125.93 mg/dl, with 10 out of 14 being diabetic on insulin or oral hypoglycaemic agents (OHA). The mean preoperative FBS in the Frey’s procedure group was 122.46 mg/dl, with seven out of 13 being known as diabetic. The mean preoperative insulin dose in the LPJ group was 15.79 units and 10 units were the mean preoperative insulin dose in the Frey’s procedure group. The trends in FBS values in the LPJ group showed a small spike in the early postoperative period (two weeks) with a p-value of >0.05, and later on, it improved over 18 months of follow-up, reaching below the preoperative values (mean 109.38) (Table 3). It may be attributed to the reduction in ductal hypertension and the role of incretin. On the contrary, the FBS levels in the Frey’s procedure group revealed a significant spike in the early postoperative period (two weeks), with a mean sugar value of 148 mg/dl and a p-value of 0.01 (Table 4).

**Table 3:** Paired T-Test of Mean Pre- and Postoperative FBS for the LPJ Group FBS: fasting blood sugar; LPJ: lateral pancreatojejunostomy; Preop: preoperative; Postop: postoperative

			T-Value	P-Value
Pair 1	Preop FBS	125.93	0.845	0.413
	Postop FBS 2 weeks	131.14		
Pair 2	Preop FBS	125.93	0.287	0.779
	Postop FBS 3 months	123.14		
Pair 3	Preop FBS	129.15	1.370	0.196
	Postop FBS 6 months	114.23		
Pair 4	Preop FBS	129.15	1.537	0.150
	Postop FBS 12 months	110.77		
Pair 5	Preop FBS	129.15	1.689	0.117
	Postop FBS 18 months	109.38		

**Table 4:** Paired T-Test of Mean Pre- and Postoperative FBS for Frey’s Procedure Group FBS: fasting blood sugar; Preop: preoperative; Postop: postoperative

			T-Value	P-Value
Pair 1	Preop FBS	122.46	4.219	0.01
	Postop FBS 2 weeks	148		
Pair 2	Preop FBS	122.46	1.689	0.117
	Postop FBS 3 months	135.62		
Pair 3	Preop FBS	122.46	1.370	0.196
	Postop FBS 6 months	134.77		
Pair 4	Preop FBS	122.46	1.097	0.294
	Postop FBS 12 months	132.38		
Pair 5	Preop FBS	122.46	0.393	0.698
	Postop FBS 18 months	126.31		

The levels stayed well above the preoperative values over 18 months of follow-up. A trend of regaining the endocrine function was found but not going below the preoperative levels over 18 months and needed a longer duration of follow-up, which was beyond the scope of this study. The trends in HbA1c showed marginal improvement in the LPJ group over a six-month follow-up period (p-value 0.008) from the preoperative levels (Table 5).

**Table 5:** Paired T-Test for Pre- and Postoperative HbA1c for the LPJ Group LPJ: lateral pancreatojejunostomy; Preop: preoperative; Postop: postoperative

		HbA1c	T-Value	P-Value
Pair 1	Preop HbA1c	6.829	0.346	0.735
	Postop HbA1c 3 months	6.871		
Pair 2	Preop HbA1c	7.008	3.172	0.008
	Postop HbA1c 6 months	6.631		

It needed further follow-up as data at 12 and 18 months were not uniformly available and hence not statistically analyzed. This may be attributed to the improved drainage, reduction in ductal hypertension, and possible incretin interplay. In Frey’s procedure group, postoperative HbA1c levels at three months revealed an increase, which can be attributed to the minor but significant loss of pancreatic tissue from the head, which continued to be on the higher side at the six-month follow-up (Table 6). Statistical significance cannot be established for this as the p-value was >0.05. Trends in mean insulin dosage showed a significant spike in the early postoperative period (two weeks) both in the LPJ (p-value 0.01) and Frey’s procedure group (0.01) (Tables 7-8). However, in the LPJ group, the insulin dose showed a reduction over the 18 months of follow-up, reaching below the mean preop insulin dose. While in the Frey’s procedure group, the postoperative insulin dose remained higher throughout the 18-month follow-up period (p-value <0.05).

**Table 6:** Paired T-Test for Pre- and Postoperative HbA1c for the Frey’s Procedure Group Preop: preoperative; Postop: postoperative

	HbA1c level		T-Value	P-Value
Pair 1	Preop HbA1c	6.623	2.047	0.063
	Postop HbA1c 3 months	6.908		
Pair 2	Preop HbA1c	6.623	1.376	0.194
	Postop HbA1c 6 months	6.946		

**Table 7:** Paired T-Test for Pre- and Postoperative Insulin Dose in the LPJ Group LPJ: lateral pancreatojejunostomy; Preop: preoperative; Postop: postoperative

			T-Value	P-Value
Pair 1	Preop insulin dose	15.79	4.196	0.01
	Postop insulin dose 3 months	20.371		
Pair 2	Preop insulin dose	16.46*	1.720	0.111
	Postop insulin dose 6 months	19.23		
Pair 3	Preop insulin dose	16.46*	2.941	0.660
	Postop insulin dose 12 months	17.23		
Pair 4	Preop insulin dose	16.46*	5.122	0.435
	Postop insulin dose 18 months	15.08		

**Table 8:** Paired T-Test for Pre- and Postoperative Insulin Dose in the Frey’s Procedure Group LPJ: lateral pancreatojejunostomy; Preop: preoperative; Postop: postoperative

			T-Value	P-Value
Pair 1	Preop insulin dose	10	4.544	0.01
	Postop insulin dose 3 months	16		
Pair 2	Preop insulin	10	4.879	0.001
	Postop insulin dose 6 months	16.77		
Pair 3	Preop insulin dose	10	4.086	0.002
	Postop insulin dose 12 months	15.69		
Pair 4	Preop insulin dose	10	3.518	0.004
	Postop insulin dose 18 months	15.08		

## Discussion

This study was focused on the endocrine outcome of two surgical treatment modalities employed for the management of CCP: LPJ and Frey’s procedure. Both procedures are similar but differ in the amount of pancreatic tissue removed and indications. Frey’s procedure is useful in clinical scenarios where the head of the pancreas is studded with calculi along with ductal hypertension and removes a variable amount of pancreatic tissue amounting to almost 20-25 % while LPJ focuses on ductal decompression through ductal deroofing [11,12].

Classically, it was proved that the drainage procedures do not alter the diabetic status either in the early postoperative or late follow-up stages [13]. Distal pancreatectomy will compromise the endocrine function without affecting the exocrine functions at an earlier stage, and proximal pancreatectomy precipitates exocrine but not endocrine insufficiency [14]. The difference is attributable in part to the relative preponderance of islet cells in the body and tail of the pancreas.

A recent study has shown that even without an operation, all patients with alcoholic (calcific) chronic pancreatitis develop both exocrine and endocrine failure within 5-10 years of the onset of disease [15]. The onset and progression of endocrine insufficiency closely parallel those of exocrine failure [16,17]. Clearly, the resection of the functionally compromised pancreas has the potential to adversely affect pancreatic function [18].

Various investigations were employed to estimate the pancreatic endocrine function, including the estimation of serum insulin, C-peptide and 24-hour urine C-peptide, and abnormal intravenous and oral glucose tolerance tests [19]. In this study, we had employed inexpensive and convenient investigation metrics like FBS and HbA1c and found that these tests adequately reflected the clinical status of the patients.

Reflecting on earlier experiences, the exocrine function remains unchanged by drainage procedures (LPJ), with there being neither deterioration nor significant improvement (p-value >0.05) [20]. Contrary to the earlier belief that procedures in the head of the pancreas do not affect the endocrine function [21], patients in the Frey’s procedure group showed a statistically significant worsening of FBS in the early postoperative period and continued to be worse over 18 months of follow-up. Their HbA1c levels also corresponded to the observation. Insulin requirement was also increased over the follow-up period. Although the trend was falling, we need close observation over later years to find out the outcome, which is beyond the scope of this study. This may be attributed to the worsened exocrine function post-Frey's procedure influencing the glycemic control of the patient. Out of six nondiabetic patients, four required insulin postoperatively to control their glycemic levels, strongly complementing the results. Out of four nondiabetic patients who underwent LPJ, all four did not require insulin therapy at a 1.5-year follow-up.

## Conclusions

LPJ has got a minimal effect on the diabetic status of nondiabetic patients. While Frey’s procedure leads to the marginal deterioration of diabetic status and increases in insulin dosage over the initial 18 months of follow-up in both diabetic and nondiabetic patients, LPJ provides marginal improvement of diabetic status and reduction in insulin dosage over 18 months of follow-up.
